# A missed opportunity: faith leaders and the HPV vaccination effort in Addis Ababa, Ethiopia

**DOI:** 10.1080/16549716.2026.2635822

**Published:** 2026-03-04

**Authors:** Wosene Berhanu, Simon Yigremachew, Claudia Hanson, Helena Avermark, Adamu Addissie, Sibylle Herzig van Wees

**Affiliations:** a Department of Global Public Health, Karolinska Institutet, Stockholm, Sweden; b Department of Holistic Development, Ethiopian Graduate School of Theology, Addis Ababa, Ethiopia; c School of Public Health, College of Health Science, Addis Ababa University, Ethiopia

**Keywords:** Vaccine hesitancy, change agents, health information, faith leaders, HPV vaccine communication

## Abstract

**Background:**

Cervical cancer causes morbidity and mortality among women worldwide, particularly in low- and middle-income countries (LMICs). Human Papillomavirus (HPV) vaccine is crucial for cervical cancer prevention, yet the vaccination rates remain suboptimal in Ethiopia. Studies identified cultural and religious factors as key barriers. While evidence suggests that faith leaders can effectively promote public health interventions, their potential role in HPV vaccination efforts has largely been overlooked and remains inadequately understood.

**Objective:**

This study aimed to explore the perspectives of faith leaders in Addis Ababa to identify factors influencing HPV vaccination among girls.

**Methods:**

This study employed qualitative methods, using in-depth interviews with purposively selected faith leaders. The faith leaders employed by the Inter-Religious Counsel of Ethiopia (IRCE) were excluded. A total of 13 faith leaders participated in the interviews. The 5C framework informed the data collection tool, and data analysis was conducted using inductive reflexive thematic analysis (RTA).

**Result:**

Faith leaders are navigating between modern medicine and their religious beliefs, face distrust of Western vaccine aid intentions and local HPV vaccine providers, and receive fragmented or inconsistent information. These challenges make it difficult for them to act as champions for the vaccination, but with clear, well-organized information, there is an opportunity to involve them more effectively.

**Conclusion:**

Faith leaders face several challenges that limit their role in promoting HPV vaccination. This study recommends providing clear, culturally relevant materials and communication strategies to support faith leaders and their communities. With these tools, faith leaders have the opportunity to engage and become effective advocates for the elimination of cervical cancer.

## Paper context

**Main findings:**

This study identified faith leaders’ perspectives and the barriers that hinder their involvement as advocates for Human Papillomavirus vaccination in Ethiopia, including religious influence, mistrust of Western actors, and gaps resulting from inconsistent information.

**Added knowledge:**

The study provides new knowledge by emphasizing the untapped potential of faith leaders in promoting vaccine uptake and underscoring the importance of engaging them through structured, culturally tailored communication.

**Global health impact for policy and action:**

This result supports strategies to integrate faith leaders into HPV vaccination efforts, strengthening trust and their active contribution towards advancing national cervical cancer elimination goals.

## Background

Cervical cancer accounts for the mortality and morbidity of thousands of women worldwide. According to the WHO, 660,000 new cases and 342,000 deaths occurred due to cervical cancer in 2022 [1]. The rates of mortality and morbidity are more severe in LMICs [2]. In Ethiopia, cervical cancer is the second most common type of cancer, following breast cancer [3]. In recognition of the high prevalence of cervical cancer in the country, Ethiopia has recently established a strategic goal to reduce cervical cancer incidence and mortality. However, women in Ethiopia still face significant barriers to action, such as a lack of sufficient information about the disease, and health system barriers to screening services [4]. Consequently, most women are diagnosed at the advanced stages of the disease, often resulting in death [5].

The HPV vaccine is an effective measure to prevent cervical cancer, and it has been introduced in many countries globally [6]. The HPV vaccine coverage varies by context. Ethiopia launched a national HPV vaccination program for girls aged 14 in 2018 [7]. Further, in 2024, the HPV vaccination guidelines were revised to expand eligibility, covering girls aged 9–14 years [8]. These recent efforts have resulted in a significant increase in HPV vaccine uptake among Ethiopian girls [8].

Despite the increasing number of vaccinated girls worldwide, vaccine hesitancy continues to pose a significant challenge to the successful and sustainable implementations of HPV vaccination strategies [8]. Vaccine hesitancy manifests differently across settings, vaccine types, and over time, often leading to significant harm through the spread of otherwise preventable diseases [9]. According to the Strategic Advisory Group of Experts (SAGEs), vaccine hesitancy is driven by multiple factors, including vaccine access, socio-cultural factors, health system factors, and other contextual influences that shape individuals’ vaccination decisions [10–12]. Furthermore, negative attitudes toward the COVID-19 vaccine have significantly contributed to the growing vaccine hesitancy in Ethiopia [13].

Studies identified cultural and religious influences as key factors affecting HPV vaccine uptake in Ethiopia [14]. Religion plays a prominent role in Ethiopian society, and faith leaders serve as key informants within this context, exerting significant influence over many aspects of people’s lives, including health practices [15,16]. Faith leaders engage with medical information and interventions to varying degrees [15]. Understanding how they balance their faith leadership with their exposure to medical information and practices is essential for contextualizing their perspectives on HPV vaccination initiatives. Previous literature has examined the relationship between religiosity and vaccine hesitancy [16]. However, despite calls for a better understanding of how faith leaders communicate and reinforce religious beliefs related to the HPV vaccine, their perspectives have not been thoroughly investigated [14]. This represents a critical gap, as faith leaders can significantly influence HPV vaccine confidence and uptake within their communities [17]. Therefore, this study aims to explore faith leaders’ perspectives on HPV vaccination for girls in Addis Ababa, Ethiopia.

To better understand the attitudes toward HPV vaccination and how to build HPV vaccine confidence, this study was guided by the 5C model [18,19], which includes Confidence, Complacency, Constraints (or Convenience), Calculation, and Collective Responsibility [20]. The model guided the study and helped to explore faith leaders’ perspectives on the HPV vaccine, aimed to identify factors influencing HPV vaccination of girls in Addis Ababa.

## Methods

### Study design

This study used a qualitative design to explore faith leaders’ perspectives on HPV vaccination for girls and to identify factors influencing the HPV vaccine confidence and uptake within the community in Addis Ababa, Ethiopia. A qualitative method was used since very little is known about faith leaders’ perspectives on HPV vaccination in Ethiopia, and this design will therefore allow for an in-depth understanding of the topic, including how religion may influence views about vaccination and can inform culturally appropriate interventions.

### Study setting

The study was conducted in Addis Ababa, the capital city of the Federal Democratic Republic of Ethiopia, with an estimated population of 5 million [21]. Addis Ababa hosts a demographically mixed population drawn from various ethnic and religious backgrounds. Amharic is the working language of the city, though other languages, including Afan Oromo, are also widely spoken [22]. According to the Central Statistics Agency, Ethiopia has a notably young population; nearly half (47%) of the Ethiopian population is under age 15 [23].

Ethiopia is regarded as one of the most religious countries in the world, with 98% of its population actively practicing their faith [23]. The two major religions in Ethiopia are Christianity and Islam. Within Christianity, the Ethiopian Orthodox Tewahedo Church and the Ethiopian Evangelical (Protestant) Churches are the two dominant branches [24]. Protestantism is one of the fastest-growing religious groups in the country, whereas the Ethiopian Orthodox Tewahedo Church has the largest number of followers [24]. Details of the participants involved in the interviews are presented in Table 1.

**Table 1.** Study participants’ information.

Characteristic	Categories	Number of participants
Denomination	The Ethiopian Orthodox Tewahedo Church	6
	Ethiopian Muslim Followers/Brothers	2
	Ethiopian Evangelical Church Mekane Yesus	1
	The Seventh-day Adventist Church in Ethiopia	1
	Evangelical Churches Fellowship of Ethiopia	1
	Ethiopian Catholic Church	2
Age range	30–39	2
	40–49	6
	>50	5
Educational status	Masters	2
	Degree	4
	Diploma	2
	Religious education	5

Addis Ababa reflects this national religious diversity and is structurally represented through the membership framework of the Inter-Religious Council of Ethiopia (IRCE). Christianity comprises various denominations, and this study included seven religious denominations. While Protestant institutions are diverse, many are organized under the Evangelical Churches Fellowship of Ethiopia (ECFE) and registered as a single denomination with the IRCE. Among these, the Ethiopian Evangelical Church Mekane Yesu (EECMY) and the Ethiopian Kale Heywet Church (EKHC) have relatively large numbers of followers compared to other Evangelical Churches registered independently, despite being part of the broader Evangelical tradition. Therefore, they are represented separately in the data collection. The denominations selected for this study were those officially registered with the IRCE and affiliated with the Bible Society of Ethiopia (BSE). Muslim religious leaders were accessed exclusively through the IRCE.

### Study participants

The study participants were selected purposefully to represent a diverse range of religious groups in the city. Inclusion criteria focused on faith leaders serving as ministers in Churches and Mosques, as their roles in teaching and leadership at the local level. To minimize bias, leaders employed by the Inter Religious Counsel of Ethiopia (IRCE) were excluded. Youth [faith] leaders were also included due to their significant influence on the spiritual lives of young people. This approach ensured a comprehensive understanding of the perspectives surrounding the discussion topic. All participants in the study were male, reflecting the predominance of male faith leaders within the context. Eight representatives were selected for interviews based on their official registration with the IRCE, while the remaining five faith leaders were contacted through the BSE. The number of participants by denomination is presented in Table 1.

### Data collection

The data were collected by using a semi-structured interview guide, which was informed by the 5C conceptual framework [20]. The interviews were conducted in Amharic, the participants’ native language, to ensure they could express themselves freely and comfortably. Interviews were recorded using an audio recorder to capture the data after participants’ consent was obtained. Each interview lasted approximately 30 minutes. Data collection took place between March and December 2024. A total of 13 faith leaders were interviewed at the IRCE office (*n* = 6), the Best Western International Hotel (*n* = 5), and other locations convenient for the faith leaders (*n* = 2). The sample size was determined by data saturation, which was reached when the last three interviews yielded no new ideas or insights beyond those already identified. All participants provided written informed consent. Anonymity and confidentiality were maintained throughout the study. Participants were informed about the purpose of the interview and their rights, including the right to withdraw at any time. The entire data collection process was conducted in accordance with the principles outlined in the Declaration of Helsinki [25]. The data collector (WB) is a PhD student with experience in qualitative research and data collection.

### Data analysis

The recordings were transcribed verbatim and translated into English, with back-translation into Amharic incorporated to ensure accuracy. In addition, the Ethiopian supervisor reviewed the translations by listening to the recordings and cross-checking the English transcripts. The data were analyzed using Braun & Clarke inductive RTA [26]. The six phases of RTA were followed in a flexible, iterative process [27,28]. The data were coded inductively, which enabled identification of themes beyond the 5C-informed interview guide, capturing the authentic perspectives of faith leaders. The researchers immersed themselves in the data by participating in interviews, translating, transcribing, and repeatedly reading the material. Initial coding was conducted in NVivo 15 to organize, code, categorize, and map the data. Coding was independently performed by the team, followed by a discussion with the supervisors. When inconsistencies occurred, consensus was reached through discussion and a second round of coding. The coding frame was refined, the data re-coded, and ultimately three main themes and 10 categories were created in close collaboration with the last author (SHvW). Researchers engaged in reflexive practices throughout the analysis, including keeping reflective notes and critically discussing these notes with the team [26].

### Reflexivity

The first author, WB, was born and raised in Ethiopia, where she trained as a nurse and worked in various health institutions. She also has 9 years of experience in Maternal, Newborn, and Child Health services. Her experience in public health research and familiarity with religious contexts helped her relate to the faith leaders and build rapport during interviews. WB is aware of the diversity of religious beliefs in Ethiopia and strives to approach the research with sensitivity to all perspectives. WB was involved in this study in data collection, translation, analysis, and report writing. Efforts were made to mitigate potential biases through rigorous methodological approaches and constant reflection on her position during the research process. These reflections were then discussed with the last author (outsider) to ensure a continuous reflective process that included both the insider and outsider perspectives. The other researcher who was involved in data coding, SY, is a medical doctor and a student at the Addis Ababa University Health Sciences Department. SY was born and raised in Ethiopia and is an Ethiopian Orthodox Christian. SHvW is an associate professor who has worked closely with the theology school in recent years on gender equality and vaccine confidence projects. She spent the past years in Ethiopia. Previously, she worked on another research project concerning the religious views on COVID-19 vaccination.

## Results

Three main themes were created from the data analysis, describing faith leaders’ perspectives on HPV vaccination: 1) Navigating modern medicine and religious beliefs towards HPV vaccination – a window of opportunity; 2) distrust of Western ambitions and perspectives, shaping doubts about school-based HPV vaccination; and 3) a fragmented source of information that hinders faith leaders from being change agents. Table 2 presents a summary of the main themes and subthemes.

**Table 2.** Themes and subthemes created through reflexive thematic analysis.

Themes	Subthemes
Navigating modern medicine and religious beliefs towards HPV vaccination – a window of opportunity	Religious bias and beliefs influence faith leaders’ stance on HPV vaccination
Distrust of Western ambitions and perspective, shaping doubts about school-based HPV vaccination	Insufficient understanding of cervical cancer and HPV vaccination
	Appreciate the HPV vaccination effort
	Concerns and skepticism toward Western vaccine aid
	Lack of trust in the school’s HPV vaccine administrators
	The HPV vaccine can cause harm
	Ethiopian HPV vaccine experts are trusted
Fragmented sources of HPV vaccination information hinder the faith leaders from being change agents	Sources of information were scattered, disorganized, and subject to seasonal fluctuations
	Information lacks depth and scientific explanation
	Dealing with rumors and misinformation

### Navigating modern medicine and religious beliefs towards HPV vaccination – a window of opportunity

#### Religious bias and beliefs influence faith leaders’ stance on HPV vaccination

Data analysis revealed that faith leaders often struggled to draw a clear line between faith practices and the use of modern medicine. While they advocated faith-based healing, they also emphasized that they do not discourage the community from seeking medical treatment.

One leader underlined:

We tell them, without rejecting worldly medication, they come to the Church, strengthen their faith, and get Holy water-*Tsebel*, at the place where Holy water is available. (O3)

Another faith leader described a recommended health-seeking pathway, stating:

So, if there are such kinds of issues [disease/cancer], the healer and the wisdom that has it is God. So, the professionals come after God. (O2)

This lack of clear demarcation among faith leaders led some to believe that diseases come from God and cannot be solved by human knowledge. Such beliefs held by leaders were reflected in the community’s attitudes and practices. One faith leader reported:

There is such a belief in the community. What they are saying is, why should they [professionals] be concerned, since it is created by God? And they remain ignorant of the science behind it. (O1)

Such issues could be miscommunicated to the community, leading to misunderstandings of the advice they received from faith leaders. Despite these challenges, faith leaders advised people to maintain both their faith and medical care.

### Distrust of Western ambitions and perspectives shaping doubts about school-based HPV vaccination

#### Insufficient understanding of cervical cancer and HPV vaccination

For some faith leaders, this interview was the first time they had heard about cervical cancer or the HPV vaccine. The information they received was not organized in a way that was easy to understand. The faith leaders pointed out that the HPV vaccination campaign has already been implemented. According to them, knowledge and awareness should come before vaccine administration. One of the faith leaders pointed out:

There is a lack of awareness, and I don’t believe enough awareness has been created in the community. Awareness work should have been done extensively before the vaccine could be administered in the community. […] there is too much focus on administering the vaccine. (E1)

Despite the lack of knowledge and understanding about the burden of the disease and its prevention, faith leaders expressed appreciation for the government’s vaccination efforts so far.

#### Concerns and skepticism toward Western vaccine aid

Faith leaders expressed skepticism, particularly concerning Western involvement in the HPV vaccine supply. According to them, the HPV vaccination program might carry an agenda beyond protecting girls from the disease. Historical incidents explaining this concern were referenced, particularly the historical relationship between Africa and the West during the colonization era, stating:

This event, which led to the ‘scrambling of Africa’, shows that the West has historically held a negative view of the continent. (O1)

In addition, the information faith leaders had about family planning, reproductive health, and rights contributed to their concerns. According to them, family planning is perceived as a deliberate attempt to reduce the population in Africa, and the HPV vaccine is viewed as potentially linked to that agenda. As one participant mentioned:

[…] the reason is that almost all these methods are hormonal. So, what does this mean? On the one hand, they encourage childbirth in their own countries; on the other hand, they preach against giving birth here [in Ethiopia] and even invest in this agenda. So, what is the reason behind this? (O1)

Another leader raised their concern about the assigned age group and sex, since the vaccination is currently exclusively accessible for girls in Ethiopia:

[…] another issue is that the vaccine focuses on young girls. Why is it being given to them? These young girls are the future of Ethiopia. (M1)

Some even voiced concern that women who got vaccinated would not be able to have more than one child or would face difficulties with childbirth. As a result, there was a lot of fear surrounding the potential implications of the HPV vaccine for women’s reproductive future.

Another concern that the faith leaders had was that the HPV vaccine is provided through foreign donations and is not produced in the countries where the girls are receiving it. As one faith leader pointed out:

For example, the [HPV] vaccine is imported and not produced by us, it came to Ethiopia as a form of aid. What about the countries that produce the vaccine? Are they using it? If not, why? It [the vaccine] must be refined; we should not be an experiment. (M2)

This reflects their fear that Ethiopians might become the subjects of a medical experiment.

#### Lack of trust in the school vaccine implementers

Trust issues surrounding the HPV vaccine were not limited to Western actors; it is extended to local Ethiopian professionals. Faith leaders also noted their concerns about the individuals and the systems that implement the HPV vaccine. They urged further studies to be conducted to ensure the safety and effectiveness of the accessible HPV vaccine in the country. Saying,

How concerned are they [vaccine implementors]? Are they the ones administering the [HPV] vaccine? Do they understand its effects, or are they just taking it from an external source without studying it properly? They must understand the vaccine well. (M1)

Faith leaders highlighted concerns about the vaccine implementers, noting that some aspects might benefit from further investigation, while others were perceived as knowingly negligent, despite being aware of the potential negative consequences for girls. As one of the faith leaders questioning:

But I’m not sure if they (the HPV vaccine implementers) have the opportunity to check other aspects, such as the quality of the vaccine or its production process. (O1)

Another shared thought on the perceived motivations behind administrators’ actions:

We view them [the vaccine implementers] in two ways. … people who approve the vaccine as a medicine … who do their jobs properly. … .and those who give the vaccine even if they know its potential impact, such as causing infertility. (M2)

This mistrust, as one faith leader highlighted, primarily stemmed from the lack of clear communication between the community and the bodies that are responsible for implementing the HPV vaccine.

#### Fear that the vaccine can cause harm

Fear that the HPV vaccine may cause harm was consistently described as a concern. Some faith leaders expressed concerns about the vaccine’s impact on fertility or its potential link to cancer. One leader said:

I am concerned that the vaccine might cause infertility. I fear that it could prevent my daughter or other family members from having children. (M1)

Another faith leader also shared his worries about the long-term impact on health:

I am worried about … the side effects. whether mothers and sisters from the past generation received the HPV vaccine and are still living healthily and peacefully, as this could help reduce my stress. (M2)

Furthermore, beyond concerns that the vaccine could affect girls’ future fertility, faith leaders also expressed fear that the vaccine itself might cause cancer. This belief stemmed from two main concerns: first, the idea that the vaccine is developed from a weakened virus; and second, the suspicion that it may have been intentionally designed to cause harm. One of the faith leaders expressed his worry, saying,

I worry that the vaccine could be a means of causing cancer. Any vaccine is made from the disease itself, so I wonder, what if the cancer-causing virus becomes stronger in the vaccines and affects girls? How much research has been done to ensure the vaccine is safe? (M1)

However, faith leaders highlighted openness toward HPV vaccination. They believed that the vaccine could be beneficial but remarked on the need for a thorough investigation to ensure it does not negatively affect future generations. They also viewed themselves as responsible for protecting the well-being of their community. As such, they believed that this investigation should be conducted by trusted Ethiopian experts within their faith community, ensuring that the vaccine is thoroughly studied and its safety properly evaluated.

#### High trust in Ethiopian researchers and experts

Rather than rejecting vaccination completely, many faith leaders emphasized the importance of involving trusted internal experts from within their communities to evaluate the vaccine’s safety and guide the public accordingly. Even though they expressed doubts and mistrust about the HPV vaccine, which is provided through foreign aid, and the intentions behind its administration, faith leaders also placed strong trust in highly qualified Ethiopian professionals, who have studied at higher levels and are known for their commitment to the country. They believe that Ethiopian professionals are more reliable. One faith leader highlighted:

[…] there are many Ethiopians who are well-educated, and they can study the issues or examine the vaccine provided by foreign aid. (O1)

Faith leaders highlighted that their intention is not to reject vaccines supported by foreign countries, but rather to ensure their safety through careful investigation by trusted local experts. This, they believed, would foster trust within the community. In this context, faith leaders underscored that health institutions, such as hospitals and health centers, are trusted by the community due to their long-standing relationships with the people. As a result, they pointed out that vaccine administration should be carried out within these trusted institutions as opposed to schools. As one leader said:

[…] if the vaccine is administered by a well-known entity, like a hospital or health center, where the community has trust, it would be more acceptable. However, administering it through campaigns at schools or workplaces raises concerns. (E1)

They highlighted that people typically seek healthcare services at facilities they know and trust. However, when vaccines are delivered directly to communities through campaigns, especially in schools or workplaces, they may be perceived as an external imposition that could lead to suspicion and reduced acceptance.

### Fragmented sources of HPV vaccination information hinder the faith leaders from being change agents

#### Fragmented and disorganized sources of information regarding HPV vaccination

The information the faith leaders accessed regarding HPV vaccination was scattered and fragmented. Accessing clear, reliable, and scientific information was difficult. They noted a variety of information sources, including mainstream media, religious organizations, health institutions, social media, and informal discussions among friends and neighbors. One of the leaders said:

I have heard about the disease, but it’s not much. There was a program at our Church where we talked about diseases related to abortion. From that program, I gained some understanding of the issue of cervical cancer. (C1)

Another leader revealed they seek medical information through web pages, such as the Mayo Clinic and the Cleveland Clinic. However, despite their efforts, they still struggled to find clear and comprehensive information on the subject. As explained by one of the leaders,

One of my reading interests is medicine. I read sources like the Mayo Clinic, the Cleveland Clinic, and Dr. Daniel, who is known for ‘Yene Tena Tube’ and other resources. I like to follow those kinds of things. (O1)

#### The information available lacks depth and scientific explanation, and its dissemination tends to fluctuate seasonally

Faith leaders noted that the information about HPV vaccination fluctuates, often linked to the vaccination campaigns. One leader indicated:

I think I have heard about it from the media and health facilities. Cervical cancer, especially recently, has been announced repeatedly in the media to encourage children to get vaccinated and to create awareness. (C1)

The leader added, ‘I think it has been a long time since I first heard about the vaccine, and I believe the HPV vaccine is implemented seasonally’. (C1)

The other leader also stated:

I did this in some previous times when people used to talk about it. However, I haven’t found a thing that could explain it to me deeply. (M2)

Additionally, despite their efforts, the available information about the HPV vaccine lacked depth and scientific explanation. One leader said:

I did this [search for information] in the past when people used to talk about it. However, I have not found anything that could explain it to me deeply. (M2)

#### Information lacks depth and a scientific explanation

Lack of clarity about the disease and the vaccine left faith leaders uncertain about how to respond to the concerns that the community raised, as one leader mentioned:

I have also seen negative comments, especially on websites like UNICEF, where people often post negative feedback in the comment section. (C2)

Furthermore, they highlighted that there is no centralized, well-organized platform to obtain comprehensive information about the HPV vaccine. One leader remarked:

I do not think there is a well-organized or formal way to respond. However, I hear about it, and since we live in the time of technology, I hear about it from various media. (O4)

The lack of formal awareness programs and the unorganized sources of information created confusion and doubt among faith leaders and within the community, preventing faith leaders from actively encouraging HPV vaccination. They indicated that they need professional explanations rather than trying to acquire information from the web or other sources. One leader stated: ‘When we Google it, it gives us limited information’ (M2).

#### Dealing with rumors and misinformation

Faith-leaders expressed concern about the information they had received, noting that the lack of organized and timely communication from trusted sources could lead to misinformation and disinformation. They pointed out that announcements about the HPV vaccine have lacked clear and consistent messaging, which has created confusion and fostered mistrust. One leader highlighted that this communication gap has significantly contributed to the spread of misinformation within the community, sharing what some community members are saying about the HPV vaccine:

They are going to make us barren, especially Africans, particularly Ethiopians; we are not to be productive or developed in various ways. Now, they are doing this through the HPV vaccine. (C1)

According to them, such misconceptions are widespread and continue to create confusion. They also underscored the importance of delivering information through appropriate and trusted channels, stating:

This highlights the importance of consistent, accurate, and timely information to address concerns and eliminate misconceptions regarding the HPV vaccine in the community. (O6)

In addition, faith leaders shared their concerns about the lack of strong information-transferring mechanisms, especially since the vaccine is new to the community. They noted that the need for accurate information extends beyond a few community members to the broader population.

This [the HPV vaccine] is something new. Therefore, more must be done by getting closer to schools, addressing spiritual leaders, and engaging with the Kebele;^1^^1^In the Ethiopian context, a Kebele is the smallest administrative unit within the government structure. It functions as a local community-level governance body, typically responsible for organizing and overseeing a population of up to 25,000 within a defined geographic area. awareness must be created. And I don’t think enough has been done yet. (AL1)

Another leader echoed similar concerns, adding:

I think the solution is for health professionals to educate faith leaders by providing them with adequate information. (O5)

Leaders repeatedly and strongly suggested the importance of providing timely, trustworthy, and in-depth information to the community, stressing that vaccination should not be rushed without prior awareness. One leader reflected:

As I understood, the HPV vaccine has just started to be administered, but awareness was not raised first. Society must know and feel responsible for saying, ‘I have to allow my child to get vaccinated’. (E1)

## Discussion

Multiple contextual factors shape the community’s response to vaccination, including distrust of Western aid rooted in colonial narratives, mistrust of the health system, and the community’s perceptions of political and governmental bodies. Additionally, the power dynamics between the funding organization and vaccine recipients require careful consideration. This study found that faith leaders often struggled to reconcile their religious beliefs with modern medicine, including HPV vaccination. Many expressed doubts about school-based HPV vaccination and mistrusted the vaccine itself. This concern is reflected in low confidence, a core component of the 5C model, which involves trust in the HPV vaccine, those delivering it, and the broader health system. They also exhibited complacency, viewing HPV and cervical cancer as distant or low-priority issues, which reduced their urgency to become involved. Lastly, the concept of collective responsibility, promoting vaccination to protect the wider community, was largely absent. While leaders valued community well-being, they had not been mobilized or supported to act as advocates for public health.

### Faith leaders navigate modern medicine and religious beliefs toward HPV vaccination, a window of opportunity

Faith leaders navigated religion and modern medicine, often struggling to show a clear demarcation between the two. They sometimes relied more on faith practices, while other times, they accepted and supported modern medicine. Previous studies suggest that the influence of faith on vaccination is complex, that religion is not inherently opposed to vaccination, and that most faith leaders do not promote anti-vaccine views [29–31]. For example, Catholic faith leaders publicly supported vaccination, emphasizing its role in protecting public health [32]. Similarly, faith leaders in Ethiopia have also shown high trust in science, support, and encouragement of vaccination efforts within their communities [15].

However, other studies have described vaccine hesitancy within a religious context, including for HPV vaccination [33]. Certain Christian groups that emphasize faith-based healing, as well as some Islamic communities, have demonstrated resistance to vaccination, and some Jewish denominations are cautious about HPV vaccination [29,34,35]. Faith-based reasoning is often cited as a justification for vaccine refusal [36–38]. This dual reliance on both faith and modern medical approaches has also been observed in other public health contexts in Ethiopia, where individuals often navigate between the two [16]. Having gained the endorsement of informed faith leaders is important to strengthen confidence in vaccine safety. Ultimately, they become crucial partners in public health efforts [39].

### Distrust of Western ambitions is shaping doubts about school-based HPV vaccination

Although vaccines have been widely accepted, recent controversies surrounding vaccination have been raised globally [40]. Vaccine hesitancy has evolved from individual reluctance to broader community-level resistance [20]. Such forms of vaccine hesitancy and resistance were observed during the COVID-19 vaccine rollout, with the case of polio vaccine resistance in Nigeria serving as another example [9,33].

Findings from this study revealed that faith leaders in Ethiopia expressed low confidence in HPV vaccination for girls, particularly due to concerns about Western involvement in the vaccine’s supply. Considering historical narratives and current discourse, faith leaders suspect that initiatives promoting the HPV vaccine may be motivated by hidden agendas. They cited historical incidents, especially the colonial relationship between Africa and Western countries, as the basis for their concern. Many referenced the Scramble for Africa, the Berlin Conference of 1884–1885, and foreign-led family-planning agendas frequently discussed online as evidence of previous Western interference in African affairs [41,42]. This suspicion is echoed by Ethiopian author Getachew Fentahun, who argues that foreign aid can serve as a means of exerting control or ‘recolonizing’ Africa [43]. Comparable skepticism has also been observed in other African countries, often rooted in the lingering effects of colonialism [44]. Furthermore, the faith leaders’ mistrust extended beyond Western involvement to the implementation of the HPV vaccination program, and also included local Ethiopian professionals, who were perceived as ‘negligent’ and unlikely to fully consider potential side effects or unintended consequences. Similar patterns were noted in other studies, which have identified mistrust in local institutions and governments as a source of vaccine hesitancy [45–47].

### Fragmented sources of HPV vaccination information hinder faith leaders from being change agents

A lack of proper, reliable information about HPV vaccination was observed as one of the major factors undermining HPV vaccine confidence in the findings. Faith leaders reported receiving information from various sources, including government media, religious institutions, and informal channels such as social media. This information is often fragmented, inconsistent, and lacks scientific clarity and depth. As a result, faith leaders struggled to gain a complete understanding of the HPV vaccine, which limited their ability to support and advocate for girls’ vaccination effectively and respond to HPV vaccine rumors circulating in the community. In the absence of clear and reliable guidance, faith leaders are more likely to rely on faith-based reasoning, which does not necessarily lead them to promote the vaccine or counter rumors. This finding aligns with global research demonstrating significant gaps in the production and transfer of vaccine knowledge [48], including specific deficiencies in HPV vaccination knowledge [49]. Rumors often emerge when the information disseminated about the issues is unclear or fragmented [50]. This makes people feel uncertain and seek additional information. In such contexts, individuals begin to probe explanations, and this probing can fuel the spread of rumors [47,51]. Studies point out that today’s information-saturated communities are bombarded with large volumes of content daily, increasing the likelihood of receiving conflicting messages [47]. The lack of clear information created confusion among faith leaders. As a result, they struggled to address community concerns about vaccine safety. Without ongoing, comprehensive public awareness campaigns, communities are unlikely to move beyond the belief that the HPV vaccine may cause unintended effects or serve a hidden agenda. Such perceptions continue to fuel skepticism [47]. Thus, this study highlighted that faith leaders explicitly request structured, authoritative information to enable them to serve as trusted community intermediaries. Therefore, developing well-structured, accurate, and contextually grounded information is essential to empowering faith leaders to serve as effective change agents in promoting HPV vaccination.

### Limitations of the study

This study highlighted valuable insights; however, certain limitations should be acknowledged. Data collection was conducted between March and December 2024, and we acknowledge the extended time frame as a limitation. Another limitation is that the study explored the faith leader’s perspective on girls’ HPV vaccination in Addis Ababa and did not capture the perspective from other regions, particularly rural areas, where religion plays a larger role. Therefore, the findings cannot be generalized nationwide, and further research is needed to examine faith leaders’ influence in diverse contexts. The other concern is that the study did not explore the theological explanations or official doctrinal positions of each denomination; as a result, it remains unclear whether the perspectives shared stem from formal theological beliefs or are influenced by personal belief, traditional, or cultural views. This distinction is significant, and future research could benefit from examining the specific theological teachings of each denomination of HPV vaccination. The study highlighted the unavailability of female faith leaders across most religious contexts, with only a few denominations, such as EECMY, ordaining women. Including women’s voices in future research will be important. Some of the interviews conducted at the hotel venue posed a risk of background noise; we acknowledge this as a limitation.

## Conclusion

This study explored the perspectives of faith leaders on HPV vaccination in Addis Ababa, Ethiopia, and identified key barriers that limit their endorsement of the HPV vaccine and their engagement in vaccination efforts. These included low confidence in the vaccine source and its promoters, fragmented and unclear information, complacency toward HPV-related risks, and difficulty in calculating benefits due to the absence of culturally and theologically appropriate materials. Given faith leaders’ influential role in shaping community attitudes, their limited involvement underscores the need for policy action that intentionally and contextually meaningfully engages them. Therefore, this study recommends developing accurate, accessible, and theologically sound information tailored to faith leaders. Clear and consistent communication strategies, grounded in trust and culturally sensitive, are essential to equip faith leaders to serve as informed advocates. Although the Ministry of Health, Ethiopia (MOHE) has a clearly defined health promotion structure within the primary health care unit [52], effective implementation requires a strong linkage with community structures, particularly faith-based institutions. Additionally, the lack of female participants revealed a significant gap and highlighted the need for policy direction to ensure gender sensitivity during the vaccine program. As a result, faith leaders will be able to serve as effective change agents in promoting HPV vaccination.

## Supplementary Material

COREQ_wosene F.docx

## Data Availability

All data, translated and analyzed data, are available from the corresponding author upon reasonable request. However, they are not publicly available to ensure confidentiality.

## References

[1] Sung H, Ferlay J, Siegel RL, et al. Global cancer statistics 2020: GLOBOCAN estimates of incidence and mortality worldwide for 36 cancers in 185 countries. CA Cancer J Clin. 2021;71(3):209–12. doi: 10.3322/caac.21660 33538338

[2] Burayu ET, Degefa BD, Zegeye ZB, et al. Incidence and predictors of mortality among cervical cancer patients in Sub-Saharan Africa: a systematic review and meta-analysis. Elsevier Inc 2025. doi: 10.1016/j.xagr.2025.100570.15 PMC12550229 41142845

[3] Bray F, Ferlay J, Soerjomataram I, et al. Global cancer statistics 2018: GLOBOCAN estimates of incidence and mortality worldwide for 36 cancers in 185 countries. CA Cancer J Clin. 2018;68:21492.394–424. doi: 10.3322/caac.21492 30207593

[4] Bogale AL, Ali JH, Ressom HW, et al. Cervical cancer prevention and control strategy in Ethiopia: key informant clinician’s perspective. 2024. doi: 10.21203/rs.3.rs-5041880/v1 3.

[5] Dereje N, Addissie A, Worku A, et al. Barriers to early diagnosis and treatment of cervical cancer in Addis Ababa, Ethiopia: qualitative study. BMJ Open. 2025;15:6. doi: 10.1136/bmjopen-2024-087792 PMC11751850 39819910

[6] Wu S, Ploner A, Alsina AM, et al. Effectiveness of quadrivalent human papillomavirus vaccination against high-grade cervical lesions by age and doses: a population-based cohort study. Lancet Reg Health Eur. 2025 Jan 5;49:101178. doi: 10.1016/j.lanepe.2024.101178 PMC11846428 39989876

[7] WHO. Ethiopia launches human papillomavirus vaccine for 14-year-old girls [Internet]. Geneva: World Health Organization; 2018 Dec 6 [cited 2025 Oct 6]. Available from: https://www.who.int/ethiopia/news/detail/06-12-2018-ethiopia-launches-human-papillomavirus-vaccine-for-14-year-old-girl

[8] WHO. The human papillomavirus (HPV) single-dose schedule elevated the total number of vaccinated girls in Ethiopia to over 13 million. The human papillomavirus (HPV) single-dose schedule elevated the total number of vaccinated girls in Ethiopia to over 13 million | WHO | Regional Office for Africa. 2025.

[9] Shehu Narrates U. The fight against polio vaccines misconception in northern Nigeria- Professor Umaru Shehu narrates. The fight against polio vaccine misconceptions in northern Nigeria- Professor Umaru Shehu narrates | WHO |regional office for Africa. 2020.

[10] Dera M, Wondimagegnehu A, Asfaw ZG. Determinants for hesitancy in human papillomavirus (HPV) vaccine uptake among schoolgirls in Jimma Town, Ethiopia. A mixed approach: quantitative and qualitative. Reprod Health. 2023;20. doi: 10.1186/s12978-023-01711-y 8. PMC10693089 38041121

[11] Cooper S, Schmidt, BM, Ryan J, et al. Factors that influence acceptance of human papillomavirus (HPV) vaccination for adolescents: a qualitative evidence synthesis. Cochrane Database Syst Rev. 2019;2019. doi: 10.1002/14651858.CD013430 PMC11998976 40232221

[12] Strategic advisory group of experts (SAGE) on immunization working group on potential contribution of human papillomavirus (HPV) vaccines and immunization towards cervical cancer elimination background document and report to SAGE 2. 18–19.

[13] Dereje N, et al. COVID-19 vaccine hesitancy in Addis Ababa, Ethiopia: a mixed-method study. BMJ Open. 2022;12:e052432. doi: 10.1136/bmjopen-2021-052432 PMC9152622 35636790

[14] Wubu A, Balta B, Cherie A, et al. Perception about human papillomavirus vaccination among middle adolescent school girls in Addis Ababa, Ethiopia 2023: qualitative study. BMC Womens Health. 2023:23. doi: 10.1186/s12905-023-02660-1 PMC10543266 37777713

[15] Yibeltal K, Workneh F, Melesse H, et al. ‘God protects us from death through faith and science’: a qualitative study on the role of faith leaders in combating the COVID-19 pandemic and in building COVID-19 vaccine trust in Addis Ababa, Ethiopia. BMJ Open. 2024;14:e071566. doi: 10.1136/bmjopen-2023-071566 PMC11043698 38653509

[16] Tiwana MH, Smith J. Faith and vaccination: a scoping review of the relationships between religious beliefs and vaccine hesitancy. BMC Public Health. 2024;24. doi: 10.1186/s12889-024-18873-4 PMC11227154 38971784

[17] Melillo S, Strachan R, O’brien CJ, et al. Effects of local faith-actor engagement in the uptake and coverage of immunization in low-and middle-income countries: a literature review. Christian Journal for Global Health. 2021;9(1):11.

[18] Nuwarda RF, Ramzan I, Weekes L, et al. Vaccine hesitancy: contemporary issues and historical background. Vaccines. 2022;10:1595. doi: 10.3390/vaccines10101595 36298459 PMC9612044

[19] Vojtek I, van Wouw M, Thomson A. Impact of COVID-19 on vaccine confidence and uptake: a systematic literature review. Hum Vaccin Immunother. 2024:20. doi: 10.1080/21645515.2024.2384180 2,9. PMC11305033 39106971

[20] Cooper S, Betsch C, Sambala EZ, et al. Vaccine hesitancy–a potential threat to the achievements of vaccination programmes in Africa. Taylor and Francis Inc; 2018. doi: 10.1080/21645515.2018.1460987.2355-7 PMC6284499 29617173

[21] Gezahegn Berhe A, Erena B, Hassen M, et al. City profile Addis Ababa SES social inclusion and energy management for informal urban settlements. [Online]. Available from: www.eiabc.edu.et

[22] Lanza E, Woldemariam H. Multilingualism and local literacy practices in Ethiopia: language contact in regulated and unregulated spaces Oslo, Addis Ababa University Press. Multiling Margins. 2014;1:74–100. doi: 10.14426/mm.v1i1.1457

[23] Central Statistical Agency (CSA) [Ethiopia] and ICF. Ethiopia Demographic and Health Survey 2016. Addis Ababa. Ethiopia, and Rockville (MD), USA: CSA and ICF; 2016.

[24] Haustein J, Malara DM, Idris AK. Religion in contemporary Ethiopia: history, politics and inter-religious relations.” [Online]. Available from: https://www.researchgate.net/publication/373523236

[25] World Medical Association Declaration of Helsinki: Ethical principles for medical research involving human subjects. American Medical Association; 2013. doi: 10.1001/jama.2013.281053.2191-2194 24141714

[26] Braun V, Clarke V. Reflecting on reflexive thematic analysis. In: Qualitative research in sport, exercise and health. Routledge Taylor and Francis Group; 2019. doi: 10.1080/2159676X.2019.1628806

[27] Braun V, Clarke V. Using thematic analysis in psychology. Qual Res Psychol. 2006;3:77–101. doi: 10.1191/1478088706qp063oa

[28] Braun V, Clarke V. One size fits all? What counts as quality practice in (reflexive) thematic analysis? Qual Res Psychol. 2021;18:328–352. doi: 10.1080/14780887.2020.1769238

[29] Muravsky NL, Betesh GM, McCoy RG. Religious doctrine and attitudes toward vaccination in Jewish law. J Relig Health. 2023;62:373–388. doi: 10.1007/s10943-021-01447-8 34708328 PMC8549591

[30] Kaaria F, Odhiambo FB, Okenyoru DS, et al. Religious leaders´ willingness to promote the uptake of human papillomavirus vaccine among their congregants in Mavoko sub-county, Machakos county, Kenya. Pan Afr Med J. 2024;48:6.8. doi: 10.11604/pamj.2024.48.141.43492 PMC11585127 39582912

[31] Ruijs WLM, Hautvast JLA, Van Ijzendoorn G, et al. How orthodox Protestant parents decide on the vaccination of their children: a qualitative study. BMC Public Health. 2012;12. doi: 10.1186/1471-2458-12-408 2. PMC3434025 22672710

[32] Guidry JPD, Naavaal S, Laestadius LI, et al. Health, beliefs, and faith: hPV vaccine uptake intent among Catholic, Evangelical, and mainline Protestant parents. Hum Vaccin Immunother. 2024;20. doi: 10.1080/21645515.2024.2425142 PMC11633157 39653069

[33] Troiano G, Nardi A. Vaccine hesitancy in the era of COVID-19. Palermo, Italy: Elsevier B.V; 2021. p. 245–251. doi: 10.1016/j.puhe.2021.02.025 PMC7931735 33965796

[34] Appiah EO, Oti-Boadi E, Appiah S, et al. Acceptance of HPV vaccination in boys among mothers from selected churches in Accra, Ghana. BMC Public Health. 2023;23. doi: 10.1186/s12889-023-16028-5 3,4. PMC10234580 37264392

[35] Al Shdefat S, Al Awar S, Osman N, et al. Health care system view of human papilloma virus (HPV) vaccine acceptability by Emirati men. Comput Math Methods Med. 2022;2022:1–11. doi: 10.1155/2022/8294058 4. PMC8816567 35126638

[36] Trangerud HA. ‘What is the problem with vaccines?’ A typology of religious vaccine skepticism. Elsevier Ltd; 2023. doi: 10.016/j.jvacx.2023.100349.4-8 PMC10362305 37484867

[37] Bank W. Trusted voices: engaging religious and community leaders to advance human papillomavirus (HPV) vaccination. Event | Trusted Voices: Engaging religious and community leaders to advance human papillomavirus (HPV) vaccination; 2025.

[38] Volet AK, Scavone C, Catalán-Matamoros D, et al. Vaccine hesitancy among religious groups: reasons underlying this phenomenon and communication strategies to rebuild trust. Front Media S A. 2022;10. doi: 10.3389/fpubh.2022.824560 PMC8858841 35198525

[39] Lahijani AY, King AR, Gullatte MM, et al. Hpv vaccine promotion: the church as an agent of change. Soc Sci Med. 2021;268:113375. doi: 10.1016/j.socscimed.2020.113375 32979772 PMC7755816

[40] Wong LP, Wong PF, Megat Hashim MM, et al. Multidimensional social and cultural norms influencing HPV vaccine hesitancy in Asia. Hum Vaccin Immunother. 2020 7;16:1611–1622. doi: 10.1080/21645515.2020.1756670 32429731 PMC7482900

[41] Kaufman CE. Reproductive control in apartheid South Africa. Popul. Stud. (NY). 2000;54:105–114. doi: 10.1080/713779059 11624288

[42] Schreuder DM. The scramble for Southern Africa, 1877–1895. Vol. 80. Cambridge: Cambridge University; 1980.

[43] Fentahun G. Foreign aid in the post-colonial Africa: means for building democracy or ensuring Western domination? 2023. Cogent OA. 2023;9:7–9. doi: 10.1080/23311886.2023.2241257

[44] Falade B. The colonial effect: language, trust and attitudes to science as predictors of vaccine hesitancy across Africa. Cultures Sci. 2024 7:98–118. doi: 10.1177/20966083241257338

[45] Schmid P, Rauber D, Betsch C, et al. Barriers of influenza vaccination intention and behavior - a systematic review of influenza vaccine hesitancy, 2005–2016. Pub Lib Sci. 2017 Jan 01;12:5–6. doi: 10.1371/journal.pone.0170550 PMC5268454 28125629

[46] Krastev S, et al. Institutional trust is a distinct construct related to vaccine hesitancy and refusal. BMC Public Health. 2023:23. doi: 10.1186/s12889-023-17345-5 PMC10714562 38082287

[47] de F Igueiredo A, Simas C, Karafillakis E, et al. Mapping global trends in vaccine confidence and investigating barriers to vaccine uptake: a large-scale retrospective temporal modelling study. Lancet. 2020 Sep;396:898–908. doi: 10.1016/S0140-6736(20)31558-0 32919524 PMC7607345

[48] Singh P, Dhalaria P, Kashyap S, et al. Strategies to overcome vaccine hesitancy: a systematic review. Syst Rev. 2022:11. doi: 10.1186/s13643-022-01941-4 4,5. PMC9044888 35473819

[49] Rosen BL, Shew ML, Zimet GD, et al. Human papillomavirus vaccine sources of information and adolescents’ knowledge and perceptions. Glob Pediatr Health. 2017;4:1779–1781. doi: 10.1177/2333794X17743405 PMC5703096 29204462

[50] Larson HJ. Negotiating vaccine acceptance in an era of reluctance. Aug. 2013;9:1779–1781. doi: 10.4161/hv.25932 PMC3906281 23896582

[51] Herzig van Wees S, Dini S. The silent shot: an analysis of the origin, sustenance and implications of the MMR vaccine–autism rumour in the Somali diaspora in Sweden and beyond. Glob Public Health. 2023;18. doi: 10.1080/17441692.2023.2257771 8. 37750434

[52] Tadesse AW, Gurmu KK, Kebede ST, et al. Analyzing efforts to synergize the global health agenda of universal health coverage, health security and health promotion: a case-study from Ethiopia. Global Health. 2021 Dec;17. doi: 10.1186/s12992-021-00702-7 PMC8074348 33902625

