# Supplementary material for: A missed opportunity: faith leaders and the HPV vaccination effort in Addis Ababa, Ethiopia
Source: Glob Health Action. 2026 Mar 4;19(1):2635822. doi: 10.1080/16549716.2026.2635822 (PMC12961706; doi:10.1080/16549716.2026.2635822)
Supplement: COREQ_wosene F.docx [file ZGHA_A_2635822_SM9915.docx]

Table 1. COREQ checklist, adapted from Tong et al. (2007).

| **Topic** | **#** | **Item description** | **p.** | |
| --- | --- | --- | --- | --- |
| **DOMAIN 1: RESEARCH TEAM AND REFLEXIVITY** | | | |  |
| *Personal characteristics* |  |  |  | |
| Interviewer/facilitator | 1 | Which author/s conducted the interview or focus group? | 7 | |
| Credentials | 2 | What were the researcher’s credentials? Eg, PhD, MD | 7 | |
| Occupation | 3 | What was their occupation at the time of the study? | 7-8 | |
| Gender | 4 | Was the researcher male or female? | 7-8 | |
| Experience and training | 5 | What experience or training did the researcher have? | 7-8 | |
| *Relationship with participants* | | | |  |
| Relationship established | 6 | Was a relationship established prior to study commencement? | 8 | |
| Participant knowledge of the interviewer | 7 | What did the participants know about the researcher? e.g. personal goals, reasons for doing the research | 6 | |
| Interviewer characteristics | 8 | What characteristics were reported about the interviewer/facilitator? e.g. Bias, assumptions, reasons and interests in the research topic | 7-8 | |
| **DOMAIN 2: STUDY DESIGN** | | |  | |
| *Theoretical framework* |  |  |  | |
| Methodological orientation and Theory | 9 | What methodological orientation was stated to underpin the study? e.g. grounded theory, discourse analysis, ethnography, phenomenology, content analysis |  | |
| *Participant selection* |  |  |  | |
| Sampling | 10 | How were participants selected? e.g. purposive, convenience, consecutive, snowball | 6 | |
| Method of approach | 11 | How were participants approached? e.g. face-to-face, telephone, mail, email | 7 | |
| Sample size | 12 | How many participants were in the study? | 9 | |
| Non-participation | 13 | How many people refused to participate or dropped out? Reasons? | 7 | |
| *Setting* |  |  |  | |
| Setting of data collection | 14 | Where was the data collected? e.g. home, clinic, workplace | 7 | |
| Presence of nonparticipants | 15 | Was anyone else present besides the participants and researchers? | 7 | |
| Description of sample | 16 | What are the important characteristics of the sample? e.g. demographic data, date | 6-7, 9 | |
| *Data collection* |  |  |  | |
| Interview guide | 17 | Were questions, prompts, guides provided by the authors? Was it pilot tested? | 7 | |
| Repeat interviews | 18 | Were repeat interviews carried out? If yes, how many? | N/A | |
| Audio/visual recording | 19 | Did the research use audio or visual recording to collect the data? | 7 | |
| Field notes | 20 | Were field notes made during and/or after the interview or focus group? | N/A | |
| Duration | 21 | What was the duration of the interviews or focus group? | 7 | |
| Data saturation | 22 | Was data saturation discussed? | 6 | |
| Transcripts returned | 23 | Were transcripts returned to participants for comment and/or correction? | NA | |
| **DOMAIN 3: ANALYSIS AND FINDINGS** | | | |  |
| *Data analysis* |  |  |  | |
| Number of data coders | 24 | How many data coders coded the data? | 7 | |
| Description of the coding tree | 25 | Did authors provide a description of the coding tree? | 10 | |
| Derivation of themes | 26 | Were themes identified in advance or derived from the data? | 7 | |
| Software | 27 | What software, if applicable, was used to manage the data? | 7 | |
| Participant checking | 28 | Did participants provide feedback on the findings? | N/A | |
| *Reporting* |  |  |  | |
| Quotations presented | 29 | Were participant quotations presented to illustrate the themes/findings? Was each quotation identified? e.g. participant number | 11-19 | |
| Data and findings consistent | 30 | Was there consistency between the data presented and the findings? | 10-19 | |
| Clarity of major themes | 31 | Were major themes clearly presented in the findings? | 10-19 | |
| Clarity of minor themes | 32 | Is there a description of diverse cases or discussion of minor themes? | 10-19 | |

**Reference**

Tong, A., Sainsbury, P., & Craig, J. (2007). Consolidated criteria for reporting qualitative research (COREQ): A 32-item checklist for interviews and focus groups. *International Journal for Quality in Health Care: Journal of the International Society for Quality in Health Care*, *19*(6), 349–357. https://doi.org/10.1093/intqhc/mzm042
